# Development and Validation of an Algorithm for the Digitization of ECG Paper Images

**DOI:** 10.3390/s22197138

**Published:** 2022-09-21

**Authors:** Vincenzo Randazzo, Edoardo Puleo, Annunziata Paviglianiti, Alberto Vallan, Eros Pasero

**Affiliations:** 1 Department of Electronics and Telecommunications, Politecnico di Torino, 10129 Turin, Italy; 2 Dipartimento di Fisica, Università di Torino & Sezione INFN di Torino, 10125 Turin, Italy

**Keywords:** digitization, electrocardiogram, ECG, heart pathologies, Pearson’s coefficient measurement, signals similarity

## Abstract

The electrocardiogram (ECG) signal describes the heart’s electrical activity, allowing it to detect several health conditions, including cardiac system abnormalities and dysfunctions. Nowadays, most patient medical records are still paper-based, especially those made in past decades. The importance of collecting digitized ECGs is twofold: firstly, all medical applications can be easily implemented with an engineering approach if the ECGs are treated as signals; secondly, paper ECGs can deteriorate over time, therefore a correct evaluation of the patient’s clinical evolution is not always guaranteed. The goal of this paper is the realization of an automatic conversion algorithm from paper-based ECGs (images) to digital ECG signals. The algorithm involves a digitization process tested on an image set of 16 subjects, also with pathologies. The quantitative analysis of the digitization method is carried out by evaluating the repeatability and reproducibility of the algorithm. The digitization accuracy is evaluated both on the entire signal and on six ECG time parameters (R-R peak distance, QRS complex duration, QT interval, PQ interval, P-wave duration, and heart rate). Results demonstrate the algorithm efficiency has an average Pearson correlation coefficient of 0.94 and measurement errors of the ECG time parameters are always less than 1 mm. Due to the promising experimental results, the algorithm could be embedded into a graphical interface, becoming a measurement and collection tool for cardiologists.

## 1. Introduction

The digitization process is an essential process for the analysis and processing of signals. In recent decades, the in-depth study of medical signals has been made possible thanks to its digital nature.

The fundamental advantages of digital signals are known in terms of security, storage, and the absence of information corruption due to paper deterioration. Furthermore, saving the patient’s history guarantees the ease of knowing his clinical evolution. Finally, knowing the entire time series of each signal, it is possible to implement some algorithms for the automatic detection of pathologies [1,2,3].

The ECG signal is an electrical signal that describes cardiac activity. The graph represents the trend of the heart potential over time. Nowadays, using the latest generation of electrocardiographs, digital signals can be collected and stored in the cloud. However, to know the patient’s medical history in depth and build automatic algorithms, it is essential to know the ECG signals of the past, which, in most cases, are paper-based. In this way, this tool, integrated into modern electrocardiographs, can be used to carry out a retrospective analysis of patients to deepen the ECG characteristics for making timely diagnoses [4], even in the case of rare diseases/syndromes, e.g., Short-QT [5,6]. In this sense, in order to preserve the patient history, it is essential to use a digitization method, i.e., a conversion of a paper image to digital data, for extracting the ECG signals. In [7], an entropy-based methodology is proposed. It relies on bit plane slicing (EBPS) where pre-processing is performed using dominant color detection and local bit plane slicing. The adaptive bit plane selection based on maximum entropy is applied to the pre-processed image. Discontinuous ECG correction (DECGC) is then performed to produce a continuous ECG signal. The algorithm is tested on 836 different degraded paper ECG records obtained from various diagnostic centers. After analysis, the RMS error and the correlation between the extracted digitized signal and the ground truth for 101 cases were 0.040 and 99.89%, respectively.

To increase the accuracy of the digitizing process, it is necessary to reduce noise with image-processing algorithms (often characterized by annotation to include some characters, for example, the kind of pathology) [8]. Asymmetry and noise are common mistakes in image scanning and should be avoided to capture better results. In [9], scanned images were enhanced by applying a skew correction operation using the Hough transformation, and noise removal was done using a median filter. Next, the grid was removed from the ECG images using color segmentation.

In this paper, a Matlab-based tool, the ECG-dig, for digitizing paper-based ECGs is presented. The conversion technique is validated by carrying out a similarity study based on the Pearson coefficient between the true digital signal and the digitized one, and by evaluating the algorithm in terms of its repeatability and reproducibility.

The rest of the paper is organized as follows. Section 2 describes the data acquisition system with signals of different cardiac conditions; it defines the methodology of the proposed algorithm for the ECG signal digitization and explains the algorithm validation technique based on similarity, repeatability, and reproducibility. Section 3 details the experimental results with a comparative analysis between the true digital signal and the digitized one. Section 4 discusses the used technique and its possible applications, followed by a conclusion in Section 5.

## 2. Materials and Methods

**Figure 1:**
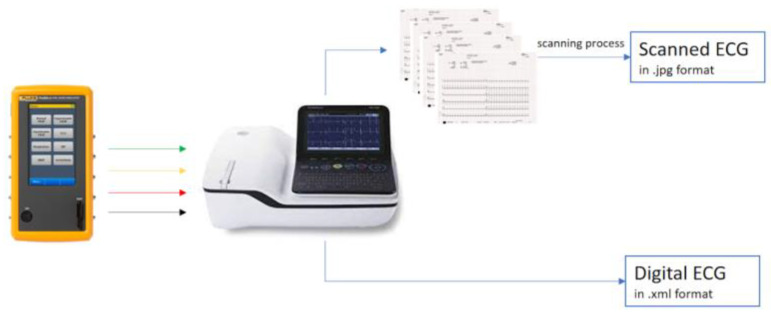
Data acquisition system. The scanning process is done by the Kyocera TASKalfa 5053ci scanner.

The proposed data acquisition system is shown in Figure 1. Two medical instruments were used in cascade to build the database for our study: the Fluke ProSim 4 Vital Signs Simulator [10] and the GE MAC 2000 electrocardiograph [11]. The ProSim 4 patient simulator allows the simulation of several cardiac conditions, while the GE MAC 2000 is an electrocardiograph that, in addition to allowing the display of the simulated conditions through the monitor, guarantees the acquisition of 12 signals (leads) simultaneously, both in digital format (*.xml file and PDF file) and by printing the ECG on graph paper [12] at 25 mm/s and 10 mm/mV. The *.xml file is automatically provided by the GE electrocardiograph; it contains the ECG digital signals as vectors of amplitudes over time, one per each ECG lead, together with the recording metadata (e.g., the sampling frequency) and the ECG parameters (e.g., HR), computed per each lead. Since the sampling frequency is 500 Hz, a 5 s signal has 2500 data points. The graph paper is thermal paper, printed about one hour before the scan. The ECG tracings resist fading for roughly 5 years [13].

The simulated electrocardiographic conditions are as follows:

- *Normal sinus rhythm*: the rhythm of a healthy heart. It means the electrical impulse from the sinus node is properly transmitted [14].
- *Bradycardia*: the presence of a slow or irregular heartbeat, less than 60 beats per minute [15].
- *Tachycardia*: the increase in the number of heartbeats per minute (heart rate) under resting conditions (more than 100 beats per minute) [16].
- *Acute Pericarditis*: the inflammation of the pericardium characterized by an accumulation of fluids in the pericardial space [17].
- *Atrial Fibrillation*: rapid and disorganized atrial activation leading to an impaired atrial function [18].
- *Atrial Flutter*: heart failure when the electrical activity in the atria is coordinated. The atria contract at a much-increased rate (more than 240 beats per minute) [19].
- *Muscle tremor artifact*: a type of movement artifact. It usually happens because the patient is trembling.
- *Breath artifact*: a typical artifact caused by patient breathing [20].
- *Premature Atrial Contractions (PACs)*: a common cardiac dysrhythmia characterized by premature heartbeats in the atria [21].
- *Premature Ventricular Contractions (PVCs)*: single ventricular impulses caused by abnormal automatism of the ventricular cells or by the presence of re-entry circuits in the ventricle [22].
- *Supra Ventricular Tachycardia*: the high-rate heart rhythm originating above the ventricle [23].
- *Ventricular Tachycardia*: the hyperkinetic arrhythmia characterized by a high ventricular rate [24].

**Figure 2:**
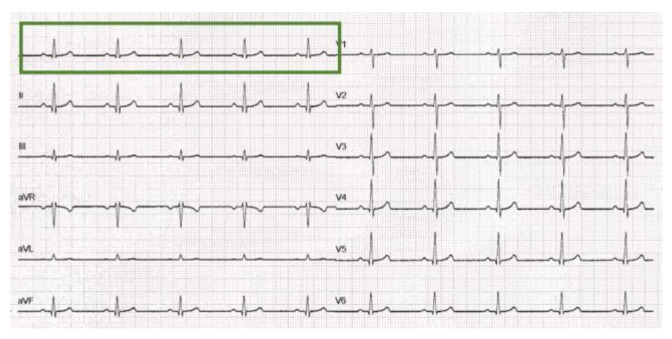
ECG of normal sinus rhythm (60 bpm); the green rectangle represents the printed portion of each lead (duration of 5 s). The sensitivity/gain is 10 mm/mV and the paper speed is 25 mm/s.

Table 1 summarizes the database artificially created with the Fluke ProSim4 simulator; 16 records have been simulated and the printed portion of each signal is 5 s long (see Figure 2).

The images printed with the electrocardiograph GE MAC 2000 were scanned with the Kyocera TASKalfa 5053ci scanner, with a scanning speed of 220 ipm and a scan resolution of 600 dpi × 600 dpi, with 256 levels of gray per color. Finally, to make the image more suitable, the contrast and sharpness were increased by 70% using the scanner software.

The purpose of the digitization algorithm is to transform the signal printed on graph paper into a digital signal that respects the measurements of millivolts (ordinate axis) and milliseconds (abscissa axis). However, in addition to the conversion error due to the digitization process, there is the error due to the electrocardiograph printing process of the signal on graph paper. In this study, the two errors are not analyzed individually, but as a combination of both.

### 2.1. The Digitization Algorithm

**Figure 3:**
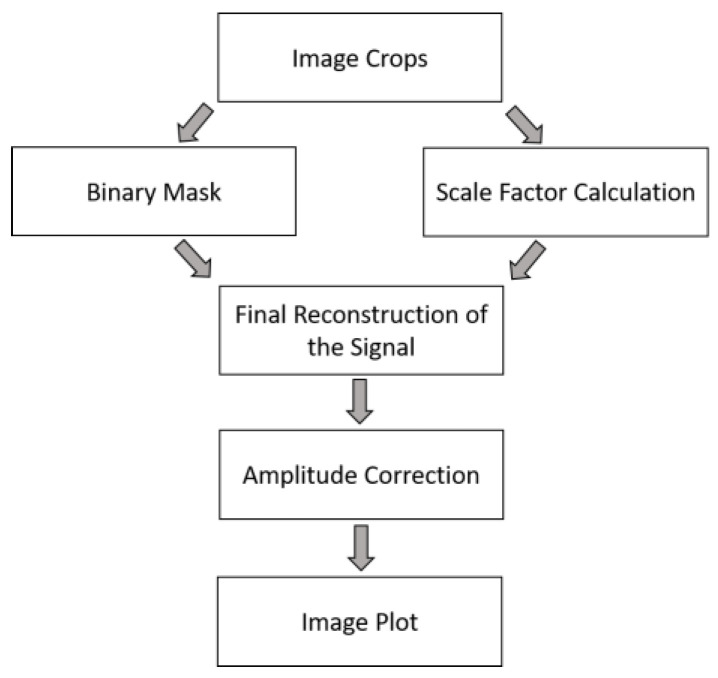
Automatic algorithm pipeline.

To extract signal data from the image, a novel algorithm was developed using the MATLAB^®^ platform. The algorithm is able to digitize the image and separate the signals from the background while respecting the time and voltage proportions of the ECGs. It is based on step-by-step automatic processing which involves the operations summarized in Figure 3.

**Figure 4:**
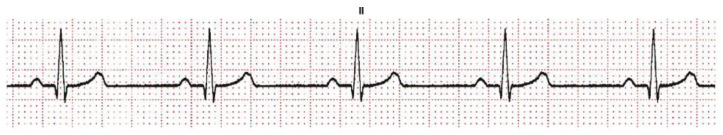
Example of the cropped image for one lead (Normal sinus rhythm, 60 bpm, II lead).

*A. Image crops*. To obtain the signals of all the ECG leads, 12 crops (one for each lead) are made on the image by framing the image patch of the corresponding lead. For each lead, the user manually makes the crops by clicking and dropping a rectangle on the portion of interest, using the “imcrop” MATLAB function (see Figure 4). Since the purpose of the proposed algorithm is to reconstruct the ECG signal respecting its morphology, independently from the number of samples, each crop does not need to have strictly the same size.

At this point, steps B and C were done in parallel.

**Figure 5:**
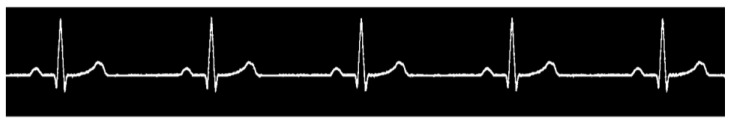
Binary mask of the cropped image (normal sinus rhythm, 60 bpm, II lead).

*B. Binary mask*. The extraction of the binary masks has been inspired by the MathWorks Community [25]. Firstly, the image is converted from RGB to HSV. Then, in order to extract the signal from the rest of the image, three ranges of color (from 0 to 1) were chosen for each HSV channel: 0.000 ≤$\;p_{H\;}$≤ 0.997; 0.000 ≤$\;p_{S}\;$≤ 0.659; and 0.647 ≤$\;p_{V}\;$≤ 1.000, where $p_{H}$, $p_{S},$ and $p_{V}$ are the values for each channel of the HSV space, within which, the pixels are considered to be part of the signal. The result is a black and white image (one per crop), as shown in Figure 5.

**Figure 6:**
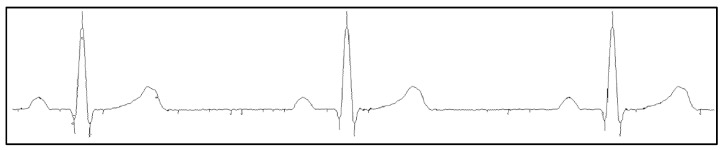
Thinned signal image (normal sinus rhythm, 60 bpm, II lead).

After, the signal is thinned (see Figure 6) using the MATLAB function “bwmorph” with the operation “shrink”, which replaces groups of neighboring pixels with a single pixel. Experimentally, it was observed that, in order to reduce noise, the best result was obtained with its parameter set to 2.5, i.e., the number of times the “shrink” operation is performed.

**Figure 7:**
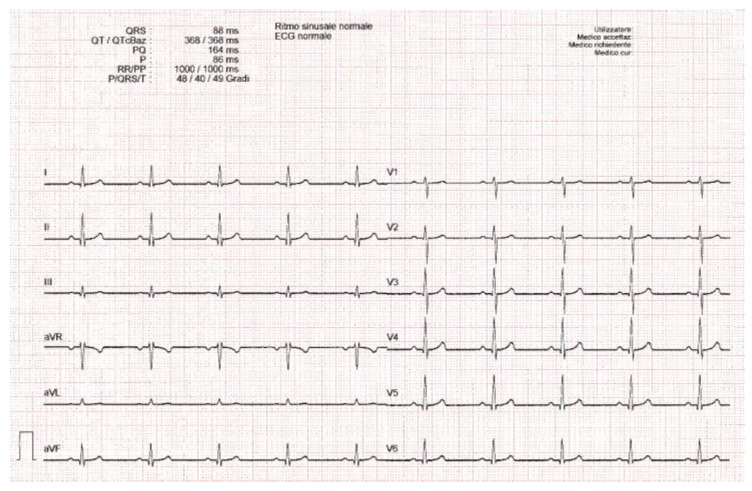
ECG leads printed on graph paper (normal sinus rhythm, 60 bpm).

*C. Scale Factor (SF) calculation*. The standard ECG leads are printed on graph paper (see Figure 7).

When the image is scanned, the correspondence between pixels and millimeters is not always the same and it depends on some factors, e.g., the printer resolution and the available type of image (scanned or PDF). Since the ECG is printed on graph paper, the grid size is fixed and known a priori. Therefore, in order to find out how much a pixel is worth in each image, a specific function was created, starting from the twelve crops, to isolate the grid and derive the scale factor.

Each crop, which is an RGB image, is transformed into a grayscale image, using the “im2gray” MATLAB function, and the signal is extracted, as in the previous paragraph, and removed from the image. For this purpose, two thresholds have been chosen quite close to the grayscale extremes in order to isolate the grid. Nevertheless, it is not certain that the remaining black points and white backgrounds have a shade of gray exactly corresponding to the extremes. Therefore, the thresholds have been chosen to be not too high (220 out of 255 for white) or low (100 out of 255 for black); in this way, black points and the white background and signal are excluded.

**Figure 8:**
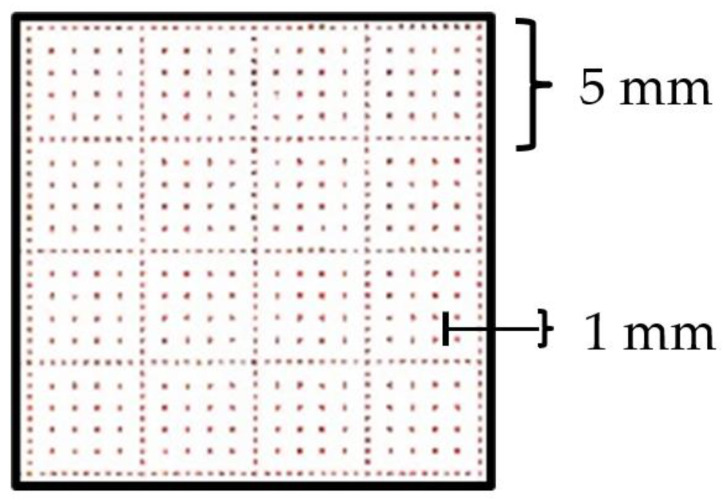
Squares in ECG graph paper.

In the proposed data set, images have two grids (see Figure 8), one less dense (with larger squares) and one denser. The first one is composed of dots, very close to each other, which form the perimeter of squares with a 5 mm side. In the second one, dots are further away and delimit squares with a 1 mm side.

**Figure 9:**
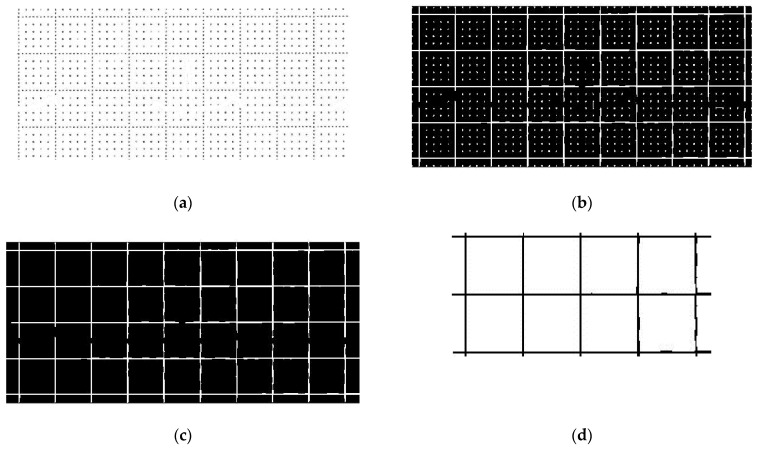
Reconstruction of the squares of the grid; (**a**) removal of the signal; (**b**) union of the nearest points; (**c**) removal of the furthest points; and (**d**) final squares with 5 mm sides.

The algorithm first searches the position of the signal points of the binary mask and transforms them into white in the original image (see Figure 9a). Then, the function “imclose” performs a morphological closing on the image in order to join the nearest points (see Figure 9b). The used structuring element object is obtained by the function “strel” with the parameters “square” (i.e., the shape of the structuring element) and “16” as the square width in pixels. To delete the furthest points, the function “regionprops” is used to return in a “struct” the property researched (“Area”) with the linear indices of the pixels in the region (“PixelIdxList”). A threshold equal to 1000 pixels was set experimentally, and below it, the areas were eliminated (see Figure 9c). In this way, we obtained a binary image with a grid, composed of horizontal and vertical lines that form 5 mm side squares (see Figure 9d). The square’s area (in pixels) is calculated as the mean of all the squares areas and, taking the square root, we have the inner side of the mean square. By summing it and the width of one line, the length *L* in pixels of the square side is found. Knowing that it should be 5 mm, the scale factor *SF* is obtained as:

*SF* = 5/*L*, (1)

where *SF* indicates how many millimeters a pixel corresponds to.

Since the shapes of the leads are different when the signal is removed, the grids show different discontinuities, which can alter the automatic recognition of the squares: in this case, identifying bigger or smaller areas, *SF* is not always the same. Therefore, the final *SF* is a mean value among the calculated *SF*s considering the 12 image crops. This one will be used in the next parts of the algorithm.

**Figure 10:**
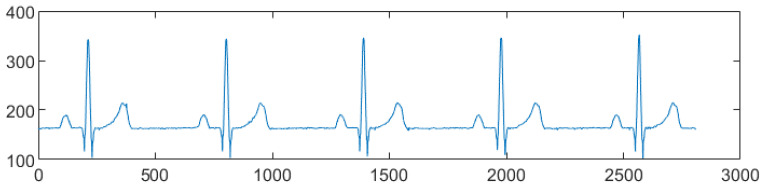
Signal reconstruction from pixels to samples (normal sinus rhythm, 60 bpm, II lead).

*D. Final reconstruction of the signal*. Once the binary images are obtained, the algorithm plots the data using the y-positions of the signal pixels as the vector of the amplitudes and joining the progressive (see Figure 10). Therefore, the amplitudes are converted from pixels to millimeters by multiplying them by *SF*.

In order to align the isoelectric line on the abscissa axis (i.e., y = 0), the most frequent amplitude value of each lead (i.e., the mode of the signal) is calculated and subtracted from the signal itself. A portion of the reconstructed signal is shown in Figure 10.

**Figure 11:**
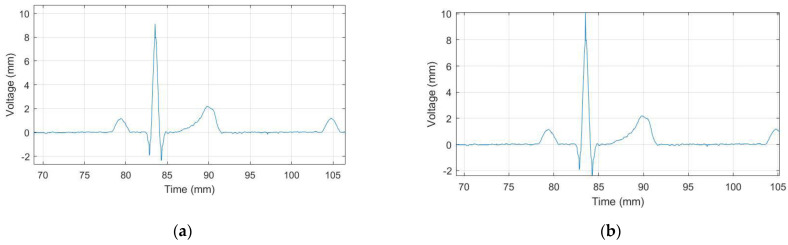
A portion of the reconstructed ECG signal (normal sinus rhythm, 60 bpm, II lead) with *SF* application; (**a**) before the amplitude correction; (**b**) after the amplitude correction.

*E. Amplitude correction*. The reconstruction by pixels leads to a pixel reduction. The result is that the amplitude is sometimes lower than reality. Furthermore, this happens where more black pixels are concentrated, especially close to R-peaks because leads are generally narrower here. Thus, the algorithm automatically detects the R-peak locations (using the “findpeaks” MATLAB function and taking the five points with the highest absolute value) and measures the peak amplitude, which is an under-estimation of the real R-peak amplitude because the image was previously processed using the “bwmorph” (shrink) operation which improves the image quality, but also introduces an error in amplitude estimation due to pixel removal. Then, it adjusts the amplitude value, adding 1 mm to those points (when the peak is positive) or subtracting 1 mm (when it is negative). We chose this quantity experimentally by averaging the differences between the reconstructed leads and the reference digital signal of some images provided by the scanner and those obtained by converting the PDF format to JPEG (as the one used in Section 2.2.3). Figure 11 shows the signal before and after the correction.

*F. Image plot*. Since 10 mm correspond to 1 mV, the signal amplitude is converted from millimeters to voltage. Regarding the time scale length, each lead has a sample number equal to the number of pixels (voltage values). In order to create the visualization of the time scale, the samples are first converted into millimeters thanks to *SF* and then in milliseconds, knowing that the paper speed is 25 mm/s.

**Figure 12:**
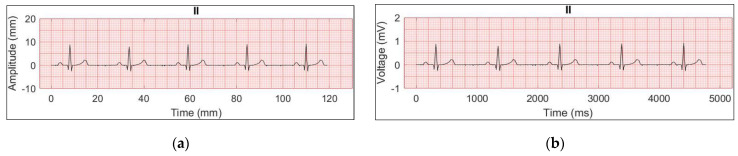
Reconstructed ECG signal (normal sinus rhythm, 60 bpm, II lead); (**a**) both axes are expressed in millimeters; (**b**) time is converted in milliseconds and voltage in millivolts (final version).

Each lead is plotted with a pink grid background which reproduces the graph paper; the x-axis is time (ms) and the y-axis is voltage (mV). An example is shown in Figure 12.

Lastly, the algorithm saves the final version of the twelve images individually (one image for each digitized lead) and as a global image with all the signals. Furthermore, it saves the voltage data of the 12 leads with the corresponding time samples.

### 2.2. Algorithm Validation Technique

The algorithm created to digitize and save the ECG signal in the digital format of each patient must be validated. As the algorithm is intended to be a tool for clinical support, it must be rigorously tested. To validate the algorithm, it was evaluated in terms of the similarity of the entire signals, using the Pearson coefficient [26], and in terms of repeatability and reproducibility, using the mean, standard deviations, range, and absolute error of the time parameters extracted from the ECG signals. In addition, the digital signal and the time parameters obtained by the *.xml file were used as references (true value). The time parameters considered, which are important because they describe the punctual behavior of the heart, are:

- R-R peak distance [27];
- QRS complex [28];
- QT interval [29];
- PQ interval [30];
- P-wave duration [31];
- Heart rate [32].

**Figure 13:**
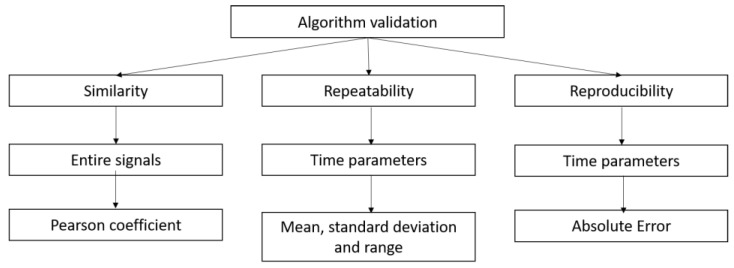
Validation scheme of the algorithm.

To assess the quality of the algorithm in terms of repeatability and reproducibility, both the time differences and the corresponding millimeters differences were considered. The former measures the relative distance from the ground truth, while the latter is more specific for the digitization process of paper-based signals. In this sense, the acceptable upper bound is 1 mm, which is the paper grid resolution. Figure 13 shows the algorithm validation scheme.

The digitized signal has a different number of samples than the digital one. Thus, to evaluate similarity, repeatability, and reproducibility in reconstructing the shape of the signal point by point, a resampling is necessary for the reconstructed leads. Two points were manually identified in the reconstructed signal (two R-peaks) and the same points were taken from the digital one. Then, the program calculated the difference between the point positions in the digital signal; the digitized signal must have the same sample number between the same two points. Finally, the distances between the points in both the signals were used as parameters for the “resample” MATLAB function. The algorithm resamples the whole reconstructed lead proportionally, using an FIR Antialiasing Lowpass Filter. The resampling rate was 500 Hz as in the digital signal.

Finally, by observing the position of the first considered point and the corresponding one in the other signal, the two leads were aligned and overlapped. In this way, a fair signal analysis can be carried out.

#### 2.2.1. Similarity

In this work, similarity indicates how close the result of the measurement of the digitized signal is to the true value, i.e., the reference samples (digital signal). To assess the validity of the algorithm in terms of similarity, the Pearson correlation coefficient (*r*) was used [33], examining the entire sequence of the digital signal and the entire sequence of the digitized one. The Pearson’s coefficient measures the statistical relationship between two continuous variables, using the covariance method [15]. It is defined as follows:

(2) $$r=\frac{n*\sum_{i=1}^{n}y_{i}*{\tilde{y}}_{i\;}-\sum_{1=1}^{n}y_{i}\sum_{1=1}^{n}\tilde{y_{i}}}{\sqrt{\left[n*\left(\sum_{1=1}^{n}y_{i}{}^{2}\right)\right]-{\left(\sum_{1=1}^{n}y_{i}\right)}^{2}]*[n*\left(\sum_{1=1}^{n}{\tilde{y_{i}}}^{2}\right)-{\left(\sum_{1=1}^{n}\tilde{y_{i}}\right)}^{2}]}},$$

where *y* is the desired output (target), 𝑦̃ is the predicted values, and 𝑛 is the total number of data. It ranges from [−1, 1]; 𝑟 = 1 indicates perfect positive correlation between *y* and 𝑦̃; 𝑟 = −1 perfect negative correlation; and 𝑟 = 0 no correlation. In our case, *y* is the reference sample from the *.xml file digital signal, while 𝑦̃ is the sample of the digitized signal.

#### 2.2.2. Repeatability

Repeatability indicates the agreement between repeated tests performed with similar measurement conditions [34,35,36]. In order to evaluate it, an image of the data set (in particular, the patient with normal sinus rhythm and 60 bpm) was chosen, and 12 lead crops were done 10 times on the same image. Since *SF* is slightly dependent on the crop made, as explained in Section 2.1.C, *SF* was collected and compared each of the ten times with the others. Trying to keep the crop shapes as similar as possible with each repetition, the variation of the most important time parameter (caused by *SF* variability) was also analyzed by calculating the mean, standard deviation, and range of each one.

#### 2.2.3. Reproducibility

**Figure 14:**
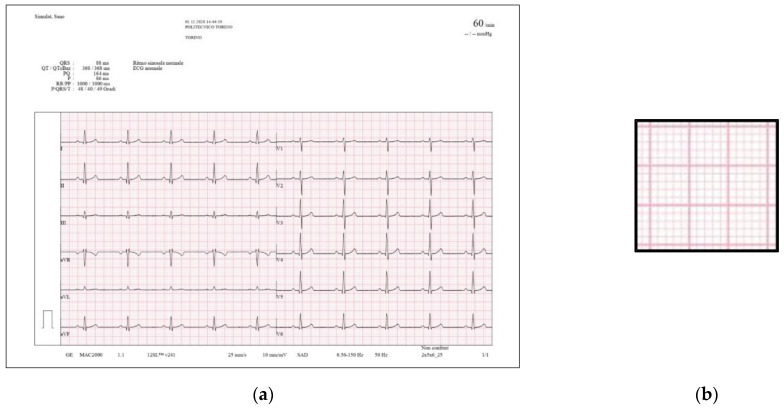
Graph paper with another format, used for reproducibility (normal sinus rhythm, 60 bpm); (**a**) PDF converted to JPEG; (**b**) structure of the grid.

Reproducibility is defined as the agreement between two measurements done under different circumstances [37,38,39]. In this case, the test was performed by using an image (the chosen patient is the same as in repeatability) with two different formats: one is the JPEG produced by the scanned electrocardiogram (see Figure 7) and the second is the PDF saved by the GE MAC 2000 and then converted in JPEG, with a different structure of graph paper where the grid is not composed by points but from solid lines (see Figure 14).

The two formats have different resolutions, the first one is 7014 × 4160 pixels, while the second is 1755 × 1240 pixels. After digitizing the two images, *SF* and the time parameters were extracted for both and compared one by one with the parameters of the digital signal by calculating the absolute Error (*aE*) which measures the difference between the measured value and the true value, computed as follows:

*aE* = | *X_experimental* − *X_true* |, (3)

where *X_experimental* is the time parameter from the digitized signal and *X_true* is the value of the digital one extracted from the *.xml file.

## 3. Results

### 3.1. Similarity

**Figure 15:**
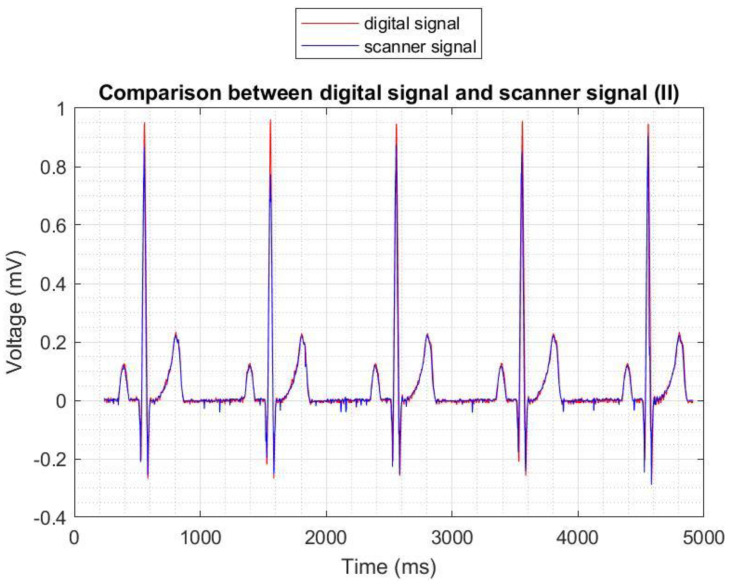
Comparison between the digitized (**blue**) and digital (**red**) signal for lead II (Normal sinus rhythm, 60 bpm).

Figure 15 shows the similarity between the signals for the normal sinus 60 bpm case (in particular for the II lead). Notably, the morphology is respected in comparison to the digital signal.

Table 2 illustrates the Pearson coefficient for each pathology, i.e., each simulated record of the dataset. If the signals are highly correlated and superimposable, the Pearson coefficient is close to 1. In the best case (normal sinus rhythm, 60 bpm) the Pearson coefficient is equal to 0.9821; in the worst case (bradycardia, 30 bpm), the Pearson coefficient is equal to 0.8798.

### 3.2. Repeatability

Regarding repeatability, the *SF* value is equal to 0.043000 ± 0.000524 mm/pixel, with a maximum variation of 0.0014 mm/pixel. Instead, the values of the time parameters, expressed with mean and standard deviations, are 102.98 ± 7.95 ms (QRS complex); 369.65 ± 11.22 ms (QT interval); 176.38 ± 9.69 ms (PQ interval); 108.75 ± 1.34 ms (P-wave duration); 1012.18 ± 12.09 ms (R-R peak distance); and 59.30 ± 0.72 bpm (heart rate).

The ranges for each parameter are 17.93 ms (QRS complex); 29.36 ms (QT interval); 24.70 ms (PQ interval); 4.18 ms (P-wave duration); and 1.98 bpm (heart rate). These variations corresponds to a difference of 0.45 mm (QRS complex); 0.73 mm (QT interval); 0.62 mm (PQ interval); and 0.10 mm (P-wave duration). The highest variation (34.04 ms for R-R peak distance) corresponds to a difference of 0.85 mm between the two tests.

Comparing the means with the true values, the worst case is for the P-wave duration, where the difference is 22.75 ms, which implies a variation of 0.59 mm. This difference derives from the manual identification of the P, Q, R, S, and T points, which is, of course, prone to a higher rate of error. For the other time parameters, the differences amount to: 14.98 ms, i.e., 0.37 mm (QRS complex); 1.65 ms, i.e., 0.04 mm (QT interval); 12.38 ms, i.e., 0.31 mm (PQ interval); 12.18 ms, i.e., 0.30 mm (R-R peak distance); and 0.70 bpm (heart rate).

All the time parameters are shown in Table 3.

### 3.3. Reproducibility

Regarding reproducibility, *SF* is 0.042285 mm/pixel for the 1st JPEG and 0.172516 mm/pixel for the 2nd JPEG. Table 4 reports the comparison of the scale factor and time parameters obtained by cropping two versions of the same ECG image.

The big variation of *SF* (0.130231 mm/pixel) is caused by the different image resolutions. Furthermore, for the 1st JPEG, the maximum *aE* of the parameters is 22.51 ms (in QRS complex), which corresponds to a difference of 0.56 mm on the graph paper. It is important to notice that the *aE* for the R-R peak distance is 4.60 ms (i.e., 0.12 mm), which means an error in heart rate calculation equal to 0.28 beats. For the other time parameters, the *aE* is 7.49 ms (QT interval); 1.62 ms (PQ interval); and 20.56 ms (P-wave duration), which correspond to a variation of 0.19 mm (QT interval); 0.04 mm (PQ interval); and 0.51 mm (P-wave duration).

In the 2nd JPEG, the *aE* is 13.21 ms (QRS complex); 0.04 ms (QT interval); 0.68 ms (PQ interval); 3.71 ms (P-wave duration); and 17.85 ms (R-R peak distance). This means that the differences in millimeters are 0.33 mm (QRS complex); 0.001 mm (QT interval); 0.02 mm (PQ interval); 0.09 mm (P-wave duration); and 0.45 mm (R-R peak distance). Therefore, for this image, the highest *aE* is found in the extraction of R-R peak distance, with a heart rate calculation that differs by 1.05 beats from the true value.

It is also noteworthy that with the 2nd JPEG, there is a closer agreement with the true value; this is probably from the fact that, although the resolution is lower, the square recognition works better since the grid is made with solid lines and not with dots.

## 4. Discussion

In the previous paragraphs, the algorithm was validated in terms of similarity, repeatability, and reproducibility.

For similarity, reconstructed signals were compared with the original digital ones by calculating the Pearson coefficient, which was always close to 1 (the perfect similarity).

Regarding repeatability, the algorithm was applied ten times on the same images and the most important time parameters (QRS complex, QT interval, PQ interval, P-wave duration, R-R peak distance, and heart rate) were extracted. In addition, the mean was close to the ground truth (less than 1 mm) and the relative range was less than 1 mm in the worst case.

Considering reproducibility, the tool has two digitized versions of the same image with different resolutions and grid structures. The time parameters extracted from the two images were compared with the original values by calculating the absolute error, which was less than 1 mm in all the cases.

In summary, the similarity test shows that the reconstructed signal had a shape very close to the original one. Repeatability and reproducibility tests showed that there was always a difference lower than 1 mm. Thus, it is acceptable considering the combination of the conversion error due to the digitization process and the error due to the electrocardiograph printing process of the signal on the graph paper. In addition, the standard paper for ECG recording presents a distance between thin lines of about 1 mm, so these errors are close to the paper’s resolution.

Future works will deal with the application of this algorithm to create a training set for a machine learning prediction system that reveals cardiac pathologies. In addition, this algorithm will be subjected to an improvement phase to be able to apply it to low-resolution images and binary images (black/white) where the distinction between signal and grid is more difficult. Finally, the amplitude correction algorithm is based on a static threshold, experimentally derived from the dataset, to better generalize this phase. In the future, a more sophisticated way of performing peak correction will be investigated.

## 5. Conclusions

This study presents a novel MATLAB-based tool for digitizing ECG graph paper. The Fluke ProSim simulator connected to the GE MAC 2000 electrocardiograph was used to generate 16 images related to different pathologies. They were scanned and then digitized by the algorithm with an automatic conversion from pixels to millimeters and, thus, to milliseconds. The proposed approach reconstructs ECGs with high values of correlation (Pearson coefficient close to 1) with respect to the original digital signal, also presenting promising results in terms of repeatability and reproducibility (measurement errors of the main parameters always less than 1 mm). Finally, the correct extraction of temporal parameters related to the digitized ECG could help doctors in detecting pathology, therefore ensuring a correct diagnosis.

In light of the carried-out analyses, the presented algorithm can be considered a good support tool for cardiologists when the ECG paper images are available without the corresponding digital data.

## Figures and Tables

**Table 1:** Simulated patient description: heart rate (HR), clinical condition, ECG amplitude, and elevation of the ST segment.

HR (bpm)	Clinical Condition	Amplitude (mV)	ST Elevation (mV)
30	Bradycardia	1	0
45	Bradycardia	1	0
60	Normal sinus rhythm	0.5	0
60	Normal sinus rhythm	1	1
60	Normal sinus rhythm	1	0.5
80	Acute Pericarditis	1	0.2
100	Normal sinus rhythm	1	1
120	Sinus Tachycardia	1	1
76	Atrial Fibrillation	1	1
82	Atrial Flutter	1	1
60	Breath artifact	1	1
60	Muscle artifact	1	1
75	Premature Atrial Contractions (PACs)	1	1
78	Premature Ventricular Contractions (PVCs)	1	1
200	Supra Ventricular Tachycardia	1	1
152	Ventricular Fibrillation	1	1

**Table 2:** Pearson coefficient for each condition.

HR (bpm)	Condition	Amplitude (mV)	ST Elevation (mV)	Pearson Coefficient
30	Bradycardia	1	0	0.8798
45	Bradycardia	1	0	0.9448
60	Normal sinus rhythm	0.5	0	0.9255
60	Normal sinus rhythm	1	1	0.9434
60	Normal sinus rhythm	1	0.5	0.9821
80	Acute pericarditis	1	0.2	0.9145
100	Normal sinus rhythm	1	1	0.9147
120	Sinus tachycardia	1	1	0.9459
76	Atrial fibrillation	1	1	0.9245
82	Atrial flutter	1	1	0.9118
60	Breath artifact	1	1	0.9684
60	Muscle artifact	1	1	0.9085
75	Premature Atrial Contractions (PACs)	1	1	0.9300
78	Premature Ventricular Contractions (PVCs)	1	1	0.9134
200	Supra ventricular tachycardia	1	1	0.9236
152	Ventricular fibrillation	1	1	0.9852

**Table 3:** Variation of scale factor and time parameters obtained by cropping the same ECG image 10 times (normal sinus rhythm, 60 bpm). The first 10 rows report the values of the ten tests. The 11th, 12th, and 13th show, respectively, the mean, standard deviation (SD), and range of the 10 tests. The last row has the values of the ProSim simulator setting (True Value).

	*SF* (mm/Pixel)	QRS Complex (ms)	QT Interval (ms)	PQ Interval (ms)	P-Wave Duration (ms)	R-R Peaks (ms)	Heart Rate (bpm)
Test 1	0.042829	96.51	360.33	183.21	108.50	1008.62	59.49
Test 2	0.043802	98.70	368.52	187.47	110.96	1031.53	58.17
Test 3	0.042374	110.17	375.72	163.28	106.78	997.49	60.15
Test 4	0.043552	113.82	386.75	167.24	109.75	1025.52	58.52
Test 5	0.042838	97.10	361.55	182.21	108.52	1008.84	59.47
Test 6	0.043690	113.59	387.39	168.35	110.10	1028.46	58.34
Test 7	0.042555	95.89	358.03	182.14	107.81	1002.18	59.87
Test 8	0.042388	110.77	376.41	162.77	106.82	999.78	60.13
Test 9	0.042932	96.74	361.41	183.75	108.76	1011.05	59.34
Test 10	0.042833	96.51	360.35	183.31	108.50	1008.65	59.49
Mean	0.043000	102.98	369.65	176.38	108.75	1012.18	59.30
SD	0.000524	7.95	11.22	9.69	1.34	12.09	0.72
Range	0.014000	17.93	29.36	24.70	4.18	34.04	1.98
True Value	-	88	368	164	86	1000	60

**Table 4:** The comparison of the scale factor and the time parameters obtained by cropping two versions of the same ECG image (normal sinus rhythm, 60 bpm). The second column shows the values of the ProSim simulator (Ground Truth). The fourth column reports the absolute errors between the first image and the true value, while the sixth column shows the absolute errors between the second image and the true value.

Parameters	True Value	JPEG Image (1st Version)	Absolute Error 1	JPEG Image (2nd Version)	Absolute Error 2
*SF* (mm/pixel)	-	0.042285	-	0.172516	-
QRS complex (ms)	88	110.51	22.51	101.21	13.21
QT interval (ms)	368	375.49	7.49	368.04	0.04
PQ interval (ms)	164	162.38	1.62	163.32	0.68
P-wave duration (ms)	86	106.56	20.56	89.71	3.71
R-R peak distance (ms)	1000	995.40	4.60	1017.85	17.85
Heart Rate (bpm)	60	60.28	0.28	58.95	1.05

## Data Availability

The data presented in this study are available on request from the corresponding author.
